# Validation of a T1 and T2 mapping software for quantitative MRI

**DOI:** 10.1186/1532-429X-18-S1-W28

**Published:** 2016-01-27

**Authors:** Sebastian Bidhult, Georgios Kantasis, Anthony H Aletras, Einar Heiberg

**Affiliations:** 1 Department of Clinical physiology, Skåne University Hospital in Lund, Lund Cardiac MR Group, Lund, Sweden; 2 Faculty of Engineering, Department of Biomedical Engineering, Lund University, Lund, Sweden; 3 School of Medicine, Aristotle University of Thessaloniki, Laboratory of Medical Informatics, Thessaloniki, Greece

## Background

T1 quantification enables measurements of myocardial extracellular volume [1] and T2 mapping may be used for detection of edema in acute myocardial infarction [2]. The purpose of this study was to develop and validate a freely available software for relaxation time map generation.

## Methods

The T1 & T2 mapping modules were implemented in Segment [3] and support the pulse sequence types shown in Table 1. T1 estimates were initialized by a lookup table search in an interval of 0-4000 ms and a 5 ms gap between entries. T2 estimates were initialized by linear regression to the signal logarithm. Relaxation time estimates were refined using a C-implementation of the Nelder-Mead Simplex method. Twelve Gadolinium-Agarose phantoms were used for validation at a 1.5T and a 3T MR-scanner (Siemens, Erlangen). Reference T1 values were determined using an Inversion Recovery (IR) Spin Echo sequence with TR = 10 seconds. Reference T2 values were determined using a Spin Echo sequence with TR = 10 seconds. Nelder-Mead Simplex optimization available in Matlab (Math Works, Natick, MA; 2013a) was used for reference relaxation time values.

**Table 1:** Table 1

	Supported Signal models	
Supported sequence-type	3-parameter models	2-parameter models
T1 Inversion-Recovery (MAGIR)	S(t) = |A (1 - B exp(-t/T1))|	S(t) = |A (1 - 2 exp(-t/T1))|
T1 Phase-Sensitive Inversion-Recovery (PSIR)	S(t) = A (1 - B exp(-t/T1))	S(t) = A (1 - 2 exp(-t/T1))
T1 Saturation Recovery	S(t) = A (1 - B exp(-t/T1))	S(t) = A (1 - exp(-t/T1))
T1 MOLLI/T1 Look-Locker MAG-images	S(t) = |A (1 - B exp(-t/T1*))|; T1 = T1*(B - 1)	not available
T1 MOLLI/T1 Look-Locker PSIR-images	S(t) = A (1 - B exp(-t/T1*)); T1 = T1*(B - 1)	not available
T2 Spin-Echo (Multi-Echo or Single-Echo)	S(t) = A exp(-t/T2) + B; B > 0	S(t) = A exp(-t/T2)
T2-prepared bSSFP	S(t) = A exp(-t/T2) + B; B > 0	S(t) = A exp(-t/T2)

Supported sequence types and signal models.

The proposed modules generated relaxation time maps from two free-breathing, single-shot bSSFP sequences. Saturation-Recovery (SR) and T2-preparation prepulses were used to create T1 & T2 weighting. Apart from T2-prepared images, a saturation prepared image was used in the T2 estimates, as previously proposed [4]. In addition, T1 was estimated with MOLLI at 1.5T using 5(3b)3 and 4(1b)3(1b)2 schemes. Acquisition times for the three bSSFP sequences were 13 min 15 sec for T1-SR, 11 sec for T1-MOLLI and 14 min 3 sec for the T2 sequence. Bias and Variability were determined using modified Bland-Altman analysis. Error percentages were computed by dividing the difference between the evaluated method and the reference with the reference value.

## Results

**Figure 1:**
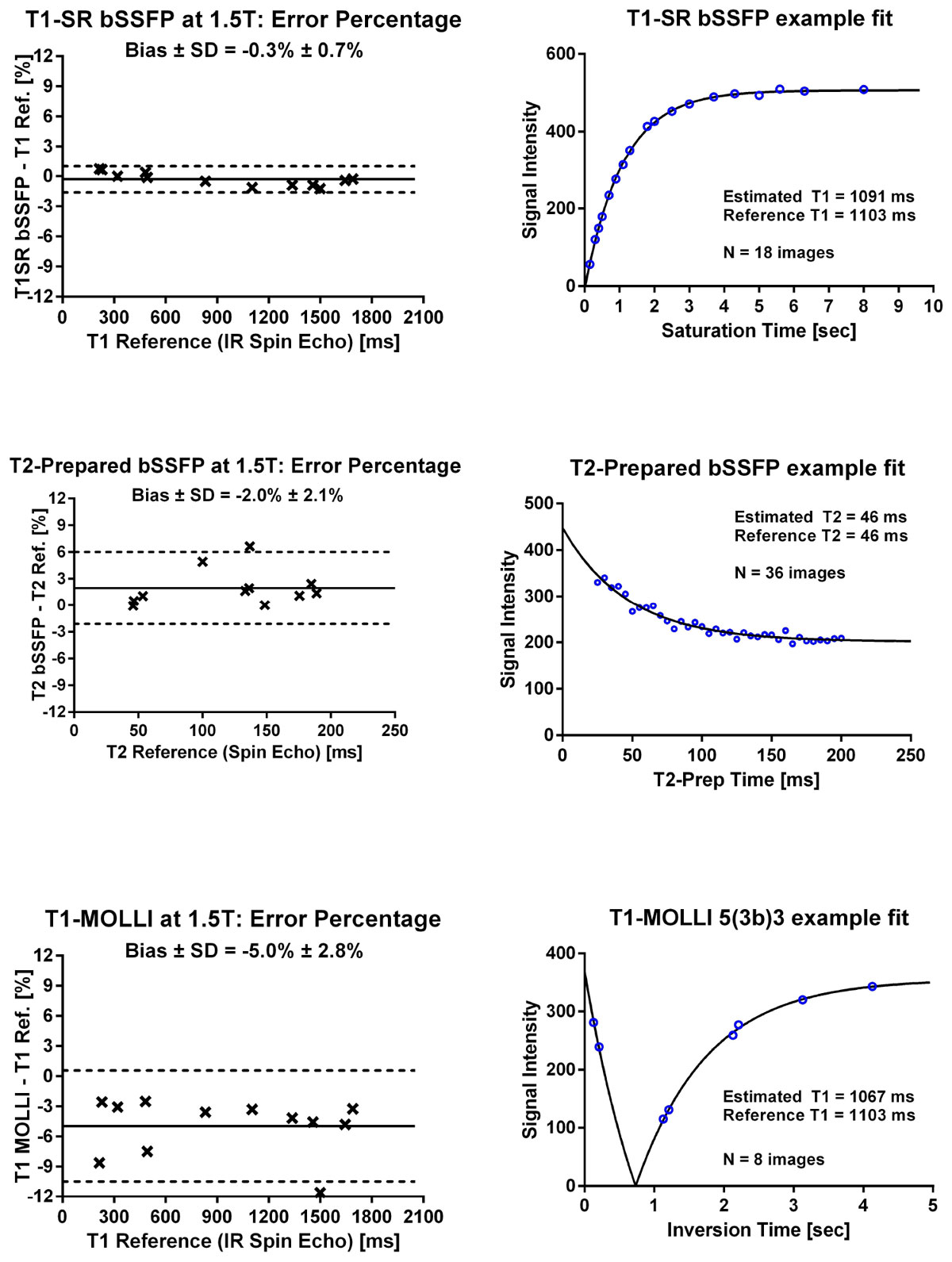
**The left panel shows modified Bland-Altman analysis of the phantom validation and the right panel shows corresponding T1 & T2 estimation examples from two phantoms**. Left panel: Crosses indicate data-points, the solid lines indicate bias and the dotted lines correspond to bias +- 1.96 SD (Standard Deviation). Right panel: Solid lines represent estimated relaxation curves and blue circles indicate data-points.

T1 and T2 reference values ranged from 214-1690 ms and 46-190 ms, respectively. Figure 1 shows the phantom validation results at 1.5T. Bias and variability (limits of agreement) at 1.5T were -0.32 ± 1.31% for SR-bSSFP and -4.96 ± 5.53% for MOLLI. MOLLI data with errors>6% originated from phantoms with reference T2 values<60 ms. Bias and variability at 3T were -2.15 ± 2.66% (SR-bSSFP). T2 bias and variability were 1.95 ± 4.06% at 1.5T and 0.37 ± 5.06% at 3T.

## Conclusions

A T1 & T2 mapping software was developed and evaluated. Low bias and variability was found for the T1-SR sequence. The increased variability of MOLLI compared to the SR sequence may be explained by the reduced number of sampling points used in MOLLI and an increasing T1 error for low T2 values. The increased variability of the T2-prepared sequence may be explained by limited SNR.
